# Renal Shear Wave Elastography for Differentiating Vasculitic and Non-Vasculitic Acute Kidney Injury †

**DOI:** 10.3390/jcm15031122

**Published:** 2026-01-31

**Authors:** Fatih Yıldırım, Samet Mutlu, Merve Sam Ozdemir, Melek Yalcin Mutlu, Alp Temiz, Sena Tolu, Gamze Akkuzu, Duygu Sevinc Ozgur, Bilgin Karaalioglu, Rabia Deniz, Gürsel Yıldız, Cemal Bes

**Affiliations:** 1 Department of Rheumatology, Başakşehir Çam and Sakura City Hospital, University of Health Sciences, 34480 İstanbul, Turkey; gamzeakkuzu89@hotmail.com (G.A.); duygu_se_a@hotmail.com (D.S.O.); bilginkaraalioglu@gmail.com (B.K.); dr.rabiadeniz@gmail.com (R.D.); cemalbes@hotmail.com (C.B.); 2 Department of Radiology, Başakşehir Çam and Sakura City Hospital, University of Health Sciences, 34480 İstanbul, Turkey; samet_m@hotmail.com (S.M.); mervesam@msn.com (M.S.O.); 3 Department of Medicine 3-Rheumatology and Immunology, Friedrich-Alexander-Universität Erlangen-Nürnberg and Uniklinikum Erlangen, 91054 Erlangen, Germany; melek.yumermutlu@uk-erlangen.de (M.Y.M.); alp.temiz@uk-erlangen.de (A.T.); 4 Department of PMR, Medipol University School of Medicine, 34214 İstanbul, Turkey; dr.sena2005@gmail.com; 5 Department of Nephrology, Başakşehir Çam and Sakura City Hospital, University of Health Sciences, 34480 İstanbul, Turkey; gursel.yildiz@sbu.edu.tr

**Keywords:** acute kidney injury, vasculitis, renal involvement, shear wave elastography, IgA vasculitis nephritis

## Abstract

**Background/Objectives:** Early identification of vasculitic acute kidney injury (AKI) is crucial for timely immunosuppression and improved renal outcomes; however, noninvasive adjunctive diagnostic tools remain limited. Renal elastography, a noninvasive technique that quantifies renal cortical stiffness, has been primarily investigated in chronic kidney disease, whereas evidence in acute kidney injury is scarce. This study aimed to evaluate the diagnostic utility of renal shear wave elastography for differentiating vasculitic from non-vasculitic AKI and to explore the association between baseline renal cortical stiffness and vasculitic renal outcomes. **Materials and Methods:** This prospective observational study included three groups: vasculitic AKI, non-vasculitic AKI, and healthy controls. Renal cortical stiffness was measured at admission using two-dimensional shear-wave elastography (2D-SWE) by radiologists blinded to clinical information. After clinicopathological confirmation of definitive diagnoses, between-group comparisons were performed and the diagnostic performance of elastography was evaluated. Additionally, in a biopsy-confirmed immunoglobulin A vasculitis nephritis (IgAVN) cohort (*n* = 12), baseline elastography measurements were examined in relation to one-year renal outcomes to explore potential prognostic associations. **Results:** The vasculitic AKI group exhibited significantly higher mean renal cortical stiffness values (9.5 ± 1.9 kPa) compared with both healthy controls (5.53 ± 0.92 kPa) and the non-vasculitic AKI group (6.61 ± 1.89 kPa) (both *p* < 0.01). Mean renal cortical stiffness demonstrated good diagnostic performance for distinguishing vasculitic from non-vasculitic AKI (AUC 0.86, 95% CI 0.73–0.97), with an optimal threshold of 6.79 kPa yielding 91% sensitivity and 72% specificity. In the prospective one-year follow-up of the IgAVN subcohort (*n* = 12), patients with unfavorable renal outcomes tended to have higher baseline renal cortical stiffness compared with those with favorable outcomes [median (min–max), 11.2 (10.8–13.3) vs. 9.1 (5.6–11.2), *p* = 0.046]. **Conclusions:** These findings suggest that renal elastography may aid in distinguishing vasculitic from non-vasculitic acute kidney injury and may provide exploratory information on the relationship between baseline cortical stiffness and renal outcomes in IgAVN.

## 1. Introduction

Establishing the etiology of acute kidney injury (AKI) is fundamental to optimizing therapeutic strategies and prognostic evaluation, particularly in disorders such as systemic vasculitis, where early recognition and timely intervention are integral to preventing progressive renal damage [1]. Renal biopsy remains the gold standard for diagnosing many acute kidney diseases; however, despite advances in biopsy techniques, the procedure still carries a risk of complications. In addition, it is not always readily accessible and may be contraindicated in certain clinical situations, such as in the presence of a bleeding diathesis [2]. These limitations have prompted clinicians to explore non-invasive methods for evaluating renal pathology. Renal elastography is a non-invasive, quantitative ultrasound technique used to assess renal cortical stiffness [3]. To date, most published clinical studies on renal elastography have focused on the assessment of renal fibrosis in chronic kidney disease [4]. In contrast, there is limited evidence in the literature regarding its utility in acute kidney injury [5]. Vasculitic AKI is characterized by glomerular and tubulointerstital inflammation, microvascular injury, and expansion of the interstitial compartment, which are pathologic changes that may theoretically increase cortical stiffness measurable by elastography [6]. This study therefore aimed to compare renal cortical stiffness between patients with vasculitic and non-vasculitic acute kidney injury using shear wave elastography.

## 2. Materials and Methods

### 2.1. Study Design and Population

A prospective observational study was conducted between April 2023 and April 2025. The study population consisted of adult patients admitted to the rheumatology and nephrology clinics with a diagnosis of AKI. Adults aged 18–60 years with no prior history of kidney disease were eligible for inclusion. At the initial evaluation, all patients underwent a comprehensive medical history and physical examination, renal function tests, urinalysis, renal ultrasonography, and renal elastography. Renal ultrasound and elastography were performed by experienced radiologists who were blinded to all clinical data. Patients with small kidneys, a solitary kidney, renal stone, or renal cysts detected on ultrasonography were excluded. When clinically indicated, further diagnostic workup was undertaken, including antinuclear antibody (ANA) testing, extractable nuclear antigen (ENA) panel assessment, anti-proteinase 3 antibody (PR3-ANCA) and anti-myeloperoxidase antibody (MPO-ANCA) testing, and renal histopathological evaluation when appropriate. Patients whose history, examination findings, or laboratory data indicated chronic kidney damage were excluded after the diagnostic workup was completed. The etiology of AKI was then determined. For patients without renal biopsy, the final diagnosis, including the confirmation of vasculitis, was established by consensus between one senior nephrologist and one senior rheumatologist, integrating clinical presentation, laboratory findings, imaging results, and follow-up data. Based on the final diagnosis, patients were categorized into two groups: vasculitic acute kidney injury (V-AKI) and non-vasculitic acute kidney injury (NV-AKI). Additionally, a group of 20 healthy adults was included as controls. This constituted the first phase of the study, during which renal elastography findings at initial presentation were compared among these three groups.

In the second phase, patients with V-AKI were prospectively followed for one year to assess the prognostic value of renal elastography. At the end of follow-up, patients with and without unfavorable renal outcomes were compared in terms of their baseline elastography measurements. To ensure group homogeneity, this analysis was restricted to patients with IgAVN, who represented the majority of the V-AKI cohort; only patients who completed the 12-month follow-up were included in this phase of the analysis. The inclusion, exclusion, and follow-up of study participants across both phases are summarized in Supplementary Figure S1.

All procedures performed in studies involving human participants were in accordance with the ethical standards of the institutional and/or national research committee and with the 1964 Helsinki Declaration and its later amendments or comparable ethical standards. The study was approved by the Ethics Committee of the İstanbul Medipol University on 30 March 2023 (decision number: 301). Informed consent was obtained from each patient.

### 2.2. Clinical Definitions

AKI was primarily defined according to the Kidney Disease: Improving Global Outcomes (KDIGO) criteria [7]. Within the KDIGO framework, urine output criteria were interpreted together with serum creatinine changes and the clinical context. In the vasculitic AKI group, in addition to the KDIGO criteria, new-onset proteinuria exceeding 150 mg/day accompanied by microscopic hematuria was considered indicative of acute renal injury in order to capture early vasculitic glomerular involvement. This extended definition was applied only in patients with clinical and laboratory findings compatible with vasculitic renal disease, and no patient was classified as AKI based on isolated urinary abnormalities alone. Unfavorable renal outcomes were assessed during the second phase of the study in patients with vasculitic AKI after a 12-month prospective follow-up and were defined as persistent proteinuria > 150 mg/day and/or an estimated glomerular filtration rate (eGFR) < 60 mL/min/1.73 m^2^ at the end of follow-up.

### 2.3. Shear Wave Elastography Protocol

All elastography examinations were performed at initial presentation, before the start of disease-specific immunosuppressive therapy. At the time of study enrollment, patients were hemodynamically stable, and none required intensive care unit monitoring. All patients fasted for at least four hours before the procedure. Initially, both kidneys were evaluated with grayscale ultrasonography to assess renal size and cortical thickness. SWE was then performed using a Philips EPIQ 5G system (Philips Medical Systems, Bothell, WA, USA) equipped with a C5-1 convex transducer (1–5 MHz). Patients were examined in the lateral decubitus position, and measurements were obtained during an inspiratory breath-hold. For each kidney, shear wave velocities were measured in the upper, middle, and lower poles. At each pole, four valid measurements were acquired and averaged, yielding a total of 12 measurements per kidney. The region of interest (ROI) was consistently placed within the renal cortex, ensuring avoidance of renal pyramids, major vessels, and excessive transducer pressure. ROI depth was adjusted on an individual basis according to patient habitus to optimize signal quality. Measurements were accepted only when the elastogram demonstrated homogeneous color filling within the ROI and absence of motion or breathing artifacts. Elastography results were displayed as color-coded elastograms and expressed as Young’s modulus (kPa). Throughout the study period, SWE examinations were performed by two experienced radiologists, each with five years of experience in SWE, with each patient being assessed once by one of them; both radiologists were blinded to the clinical data. On average, each examination—including image acquisition and post-processing—required approximately 20 min. Only technically reliable measurements were included in the analysis. The shear wave elastography protocol was designed in accordance with previously published renal shear wave elastography methodologies [8,9].

### 2.4. Statistical Analysis

Statistical analyses were conducted using R version 4.5.1 (R Foundation for Statistical Computing, Vienna, Austria). Continuous variables were summarized as means with standard deviations, and categorical variables as counts with percentages. Baseline characteristics were first examined descriptively and then compared formally across groups. For continuous outcomes, differences among the three study groups (vasculitic AKI, non-vasculitic AKI, and healthy controls) were assessed using Welch’s analysis of variance, which is robust to unequal variances and sample sizes. Effect sizes were quantified using omega squared (Ω^2^) with 95% confidence intervals. When the global test indicated significant between-group differences, pairwise contrasts were evaluated using Games–Howell post hoc comparisons. Categorical variables were analyzed using Pearson’s chi-square test of independence, and the strength of association was summarized using Cramer’s V with corresponding confidence intervals. Correlations between continuous elastography measurements—particularly left and right kidney stiffness—were assessed using Pearson’s correlation coefficients with 95% confidence intervals. Both frequentist inference and Bayesian estimation were applied to evaluate the robustness of observed associations. To examine the discriminatory ability of renal elastography in differentiating vasculitic from non-vasculitic renal injury, receiver operating characteristic (ROC) curve analysis was performed. The optimal cut-point was determined using the Youden index, and the corresponding sensitivity and specificity were reported. Graphical displays were generated to visualize group distributions, effect sizes, and diagnostic performance. In addition, multivariable linear regression analysis was performed to assess the adjusted association between vasculitis and mean renal cortical stiffness.

A sample size calculation was performed using G*Power (version 3.1) [10]. In the absence of prior adult data on renal shear-wave elastography in vasculitic versus non-vasculitic acute kidney injury at the time of study planning, a conservative standardized effect size of Cohen’s d = 1.0 was assumed, informed by the closest available prospective renal elastography evidence in acute inflammatory glomerular disease [11]. With a two-sided α of 0.05, the required sample size was 17 participants per group for 80% power and 23 participants per group for 90% power.

## 3. Results

### 3.1. Patient Population

The study enrolled 60 adult participants, including 22 patients with vasculitic AKI, 18 patients with non-vasculitic AKI, and 20 healthy controls. Immunoglobulin A vasculitis (IgAV) was the predominant etiology in the vasculitic AKI group (14 patients, 63%). In the non-vasculitic AKI group, prerenal AKI (6 patients, 33%) and drug-associated tubulointerstitial nephritis (5 patients, 27%) accounted for the majority of cases. Renal biopsy was performed in 14 of the 22 patients in the vasculitic AKI group and in 7 of the 18 patients in the non-vasculitic AKI group. In all biopsied cases, the histopathological findings were consistent with the corresponding clinical manifestations. Detailed histopathological findings of the biopsied vasculitic AKI patients, together with their corresponding mean renal cortical stiffness values, are provided in Supplementary Table S2. In the remaining patients, the etiology of AKI was determined based on clinical and laboratory findings. The etiological causes of AKI among the included patients are presented in Table 1.

### 3.2. Baseline Patient Characteristics and Between-Group Comparisons

The overall cohort (N = 60) had a mean age of 38 ± 12 years, with comparable distributions across the vasculitic AKI (39 ± 12 years), non-vasculitic AKI (41 ± 14 years), and healthy control groups (36 ± 11 years) (*p* = 0.41). The proportion of females was also similar among the groups (50% vs. 56% vs. 55%, respectively; *p* = 0.92). Body mass index did not significantly differ between groups (*p* = 0.08).

Anatomical kidney features were evaluated using renal ultrasonography. The mean left kidney craniocaudal length was shorter in the healthy group (104 ± 11 mm) than in the two AKI groups (vasculitic AKI: 111 ± 10 mm; non-vasculitic AKI: 117 ± 10 mm) (*p* < 0.01), although all measurements remained within normal population ranges. The remaining anatomical renal measurements did not differ between the groups.

Marked differences were observed in renal function parameters. Serum creatinine levels were significantly higher in the non-vasculitic AKI group (2.66 ± 1.53 mg/dL) compared with both the vasculitic AKI (1.43 ± 1.69 mg/dL) and healthy control groups (0.63 ± 0.15 mg/dL) (*p* < 0.01). Correlating with creatinine levels, glomerular filtration rate (GFR) was lowest in the non-vasculitic AKI group (35 ± 25 mL/min/1.73 m^2^), intermediate in the vasculitic AKI group (85 ± 41 mL/min/1.73 m^2^), and highest in healthy controls (129 ± 5 mL/min/1.73 m^2^) (*p* < 0.01).

**Figure 1:**
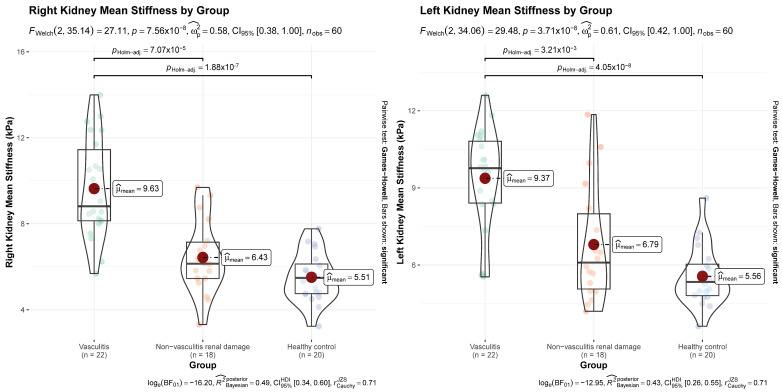
Group-wise distribution of right and left kidney cortical stiffness values. Violin plots show the distribution of measurements. Colored dots represent individual measurements, and red dots indicate mean values. Green indicates vasculitic AKI, orange non-vasculitic AKI, and violet healthy controls.

Renal cortical stiffness values differed significantly across the study groups for both kidneys. Right kidney stiffness showed a strong group effect (F(2, 35.1) = 27.1, *p* < 0.01, Ω^2^ = 0.58). Post hoc comparisons demonstrated that stiffness was significantly higher in the vasculitic AKI group compared with healthy controls (*p* < 0.01) and with the non-vasculitic AKI group (*p <* 0.01). No significant difference was observed between the non-vasculitic AKI group and healthy controls (*p* = 0.15). Left kidney stiffness also differed markedly between groups (F(2, 34.1) = 29.5, *p* < 0.01, Ω^2^ = 0.61). Similar to right kidney findings, stiffness was higher in the vasculitic AKI group relative to healthy controls (*p* < 0.01) and non-vasculitic AKI (*p* < 0.01), while the non-vasculitic AKI group did not differ from healthy individuals (*p* = 0.12). Group-wise comparisons of clinical characteristics and ultrasonographic/elastographic measurements are presented in Table 2, and the distribution of right and left kidney cortical stiffness values is depicted in Figure 1.

### 3.3. Correlation and Diagnostic Performance of Renal Cortical Stiffness

**Figure 2:**
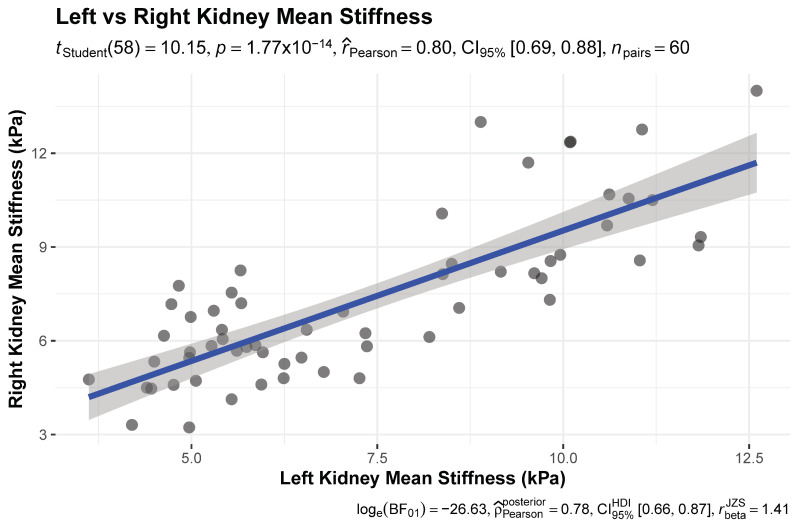
Correlation Between Left and Right Kidney Cortical Stiffness.

There was a strong positive correlation between left and right kidney cortical stiffness (Pearson’s r = 0.80, 95% CI 0.69–0.88, t (58) = 10.2, *p* < 0.001). Bayesian analysis confirmed this association (posterior ρ = 0.78, 95% HDI 0.68–0.87, BF_10_ ≈ 3.7 × 10^11^) (Figure 2).

**Figure 3:**
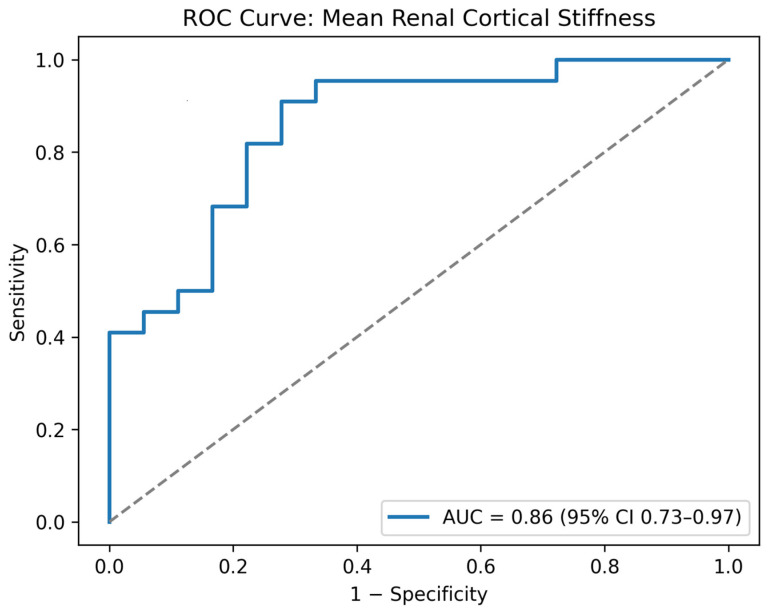
ROC curve of mean renal cortical stiffness in differentiating vasculitic from non-vasculitic AKI.

To evaluate the diagnostic performance of renal elastography, ROC analysis was conducted using the mean of left and right cortical stiffness values. This analysis demonstrated good discriminatory ability, with an area under the curve (AUC) of 0.86 (95% CI: 0.73–0.97; *p* < 0.01) (Figure 3). Using the Youden index, the optimal cut-off was 6.79 kPa, yielding a sensitivity of 91% (95% CI: 72–97%) and a specificity of 72% (95% CI: 49–88%) for distinguishing vasculitic AKI from non-vasculitic AKI. In a multivariable linear regression model adjusting for left kidney length, body mass index, and baseline serum creatinine, vasculitic AKI remained independently associated with higher mean renal cortical stiffness (β = +3.13 kPa, 95% CI: 1.68–4.58; *p* < 0.001) (Supplementary Table S3).

### 3.4. Comparison of Clinical and Elastographic Parameters by Long-Term Renal Outcome in IgA Vasculitis

Of the 14 patients with IgA vasculitis nephritis (IgAVN) identified at baseline, 12 completed the 12-month follow-up and were included in the renal outcome analysis. Age, serum creatinine, proteinuria levels, and body mass index at the first visit did not differ significantly between patients with unfavorable renal outcomes (*n* = 3) and those with favorable outcomes (*n* = 9) (all *p* > 0.05). In contrast, renal cortical stiffness values tended to be higher in patients with unfavorable outcomes. These patients exhibited higher left (median, 11.2 kPa vs. 9.6 kPa, *p* = 0.06) and right (median, 12.3 kPa vs. 8.5 kPa, *p* = 0.049) kidney cortical stiffness. When the mean stiffness of both kidneys was considered, values remained elevated in the unfavorable outcome group (median, 11.2 kPa vs. 9.1 kPa, *p* = 0.046). Clinical and elastographic characteristics according to long-term renal outcomes in IgA vasculitis are summarized in Table 3. During the 12-month follow-up period, immunosuppressive treatment characteristics were recorded, and these data are summarized in Supplementary Table S1. Given the limited number of patients in the unfavorable outcome group, these findings should be interpreted as exploratory.

## 4. Discussion

In this study, we evaluated the role of 2D-SWE in differentiating vasculitic from non-vasculitic acute kidney injury. In addition, we explored the association between baseline renal cortical stiffness and long-term renal outcomes in patients with IgAVN. Patients with vasculitic AKI exhibited significantly higher baseline renal cortical stiffness values compared with both non-vasculitic AKI patients and healthy controls, and among patients with IgAVN, those who developed unfavorable long-term renal outcomes had higher baseline cortical stiffness values at presentation. Together, these findings suggest that 2D-SWE may be useful for differentiating vasculitic AKI from other etiologies, while higher baseline cortical stiffness may be associated with unfavorable renal outcomes in IgAVN.

The use of tissue elasticity measurements and elastography techniques for the evaluation of renal pathologies has been explored since the 1990s [12,13]. Although renal elastography has been investigated across a broad spectrum of renal disorders, its clinical applications to date have focused predominantly on the diagnosis and staging of chronic kidney disease [8,14,15]. In contrast, despite ongoing technical advances in elastography, clinical studies specifically evaluating shear wave elastography in acute kidney injury remain scarce. Xu et al. first reported that alterations in renal shear wave elastography were associated with the presence of AKI in a prospective observational pilot study, demonstrating increased renal stiffness compared with controls; however, etiologic differentiation and prognostic implications were not addressed, and the cohort consisted of heterogeneous critically ill patients [16]. Subsequently, Qiang et al. evaluated shear wave elastography in critically ill patients and showed that renal stiffness differed between AKI and non-AKI states, supporting feasibility in acute settings, although AKI etiologies were not analyzed separately and longitudinal outcomes were not assessed [17]. More recently, Wang et al. combined contrast-enhanced ultrasound with elastography to predict histopathology and renal recovery in AKI, providing important insights into the prognostic potential of imaging-based renal stiffness; nevertheless, immune-mediated and vasculitic forms of kidney injury were not specifically examined [18]. In contrast to these investigations, the present study focused on vasculitic AKI, directly compared vasculitic and non-vasculitic etiologies, and evaluated the prognostic relevance of baseline 2D-SWE measurements in IgAVN, thereby extending existing evidence through an integrated etiologic and outcome-based assessment.

From a pathophysiological perspective, acute kidney injury is characterized by dynamic and potentially reversible changes, including acute interstitial edema, inflammatory cell infiltration, endothelial dysfunction, and microvascular injury, all of which can alter the mechanical properties of the renal cortex [19]. In immune-mediated forms of AKI, such as vasculitic AKI, these processes are further amplified by immune complex deposition, complement activation, and small-vessel vasculitis, leading to expansion of the interstitial compartment and increased tissue stiffness [20,21]. Such acute inflammatory and edematous changes provide a plausible biological basis for the observed increase in renal cortical stiffness detected by shear wave elastography in vasculitic AKI. Supporting this concept, studies in renal transplantation have demonstrated that shear wave-based elastography can detect increased cortical stiffness during episodes of acute allograft rejection, a condition similarly characterized by acute immune-mediated inflammation, interstitial edema, and microvascular injury [22,23]. Importantly, these elastographic changes have been shown to precede or parallel histopathological evidence of rejection, suggesting that shear wave measurements are sensitive to acute inflammatory alterations rather than chronic fibrotic remodeling alone [24]. In this context, our findings extend these observations to native kidneys affected by vasculitic AKI, indicating that 2D-SWE may capture shared pathophysiological mechanisms underlying acute immune-mediated renal injury across different clinical settings. Similarly, alterations in renal cortical stiffness have also been reported in glomerular diseases characterized by inflammatory and immune-mediated injury. Studies employing shear wave-based elastography in glomerulonephritis have demonstrated increased cortical stiffness in association with histopathological features such as interstitial inflammation, cellular infiltration, and expansion of the tubulointerstitial compartment, rather than chronic fibrosis alone [25,26]. Although these observations are not derived from cohorts specifically comprising glomerulonephritis-associated acute kidney injury, they support the concept that shear wave elastography is sensitive to inflammatory and edematous changes within the renal cortex, providing a plausible mechanistic explanation for the increased stiffness observed in vasculitic forms of kidney injury in the present study.

From a clinical perspective, the most relevant finding of the present study was the significant difference in baseline renal cortical stiffness between vasculitic and non-vasculitic AKI. Notably, this distinction was observed despite the marked etiologic heterogeneity within the non-vasculitic AKI group, suggesting that 2D-SWE captures features specific to immune-mediated renal injury rather than nonspecific impairment of renal function. Importantly, patients with non-vasculitic AKI exhibited higher serum creatinine levels and lower estimated glomerular filtration rates at presentation, indicating more pronounced functional renal impairment in this group. However, renal cortical stiffness values in the non-vasculitic AKI group were numerically higher than those observed in healthy controls but did not reach statistical significance. This observation is consistent with prior insights, as highlighted by Barr and colleagues, emphasizing that shear wave elastography reflects a complex interplay of tissue inflammation, interstitial edema, and microvascular alterations rather than renal dysfunction alone [27]. Accordingly, non-vasculitic forms of AKI, particularly those driven by prerenal mechanisms or transient tubular injury, may present with substantial functional impairment without a consistent increase in cortical stiffness. In contrast, the higher cortical stiffness observed in vasculitic AKI despite relatively better baseline renal function suggests that shear wave elastography preferentially reflects underlying inflammatory and edematous tissue changes rather than the severity of functional decline per se. In routine clinical practice, where the initial evaluation of AKI often relies on nonspecific laboratory and imaging findings, this capability may be particularly valuable for raising early suspicion of vasculitic involvement. Furthermore, the association between higher baseline renal cortical stiffness values and unfavorable long-term renal outcomes in patients with IgAVN suggests that 2D shear wave elastography may contribute to early risk stratification at the time of presentation. Although based on a limited sample size, these exploratory findings indicate that patients with unfavorable renal outcomes tended to have higher baseline proteinuria levels; however, this difference did not reach statistical significance. Given that this subgroup also exhibited higher renal cortical stiffness values, this observation may point to a potential relationship between baseline proteinuria and increased cortical stiffness. If confirmed in larger prospective cohorts, such associations could support the use of elastography-derived parameters as adjunctive markers for identifying patients who may benefit from closer monitoring or more intensive immunosuppressive therapy in vasculitic AKI.

Several limitations of the present study should be acknowledged. First, although the sample size was sufficient to allow comparison between vasculitic and non-vasculitic AKI groups, it may have been limited for prognostic assessment, particularly with respect to long-term renal outcomes. Second, both the vasculitic and non-vasculitic AKI groups exhibited etiologic heterogeneity; however, this reflects real-world clinical practice, where AKI frequently presents with diverse underlying causes. Importantly, the ability of 2D-SWE to differentiate vasculitic from non-vasculitic AKI despite this heterogeneity supports the potential clinical utility of the technique. Third, renal biopsy was not performed in approximately one-third of patients with vasculitic AKI due to clinical contraindications or patient-related factors, which precluded a systematic correlation analysis between renal cortical stiffness and histopathological findings across the entire cohort. Additionally, the lack of universally accepted and standardized histopathological activity scoring systems applicable across different forms of vasculitic renal involvement also limited the feasibility of meaningful correlation analyses. Fourth, elastography measurements were obtained at a single time point at presentation, and longitudinal changes in renal stiffness during treatment and follow-up were not evaluated. In addition, formal interobserver and intraobserver variability analyses were not performed, as elastography measurements were obtained at a single predefined time point within the framework of routine clinical assessment at presentation in a prospective observational design. In patients with suspected vasculitic AKI, prompt initiation of immunosuppressive therapy was clinically indicated following baseline evaluation, and treatment-related changes in renal inflammation, interstitial edema, and microvascular dynamics could substantially influence shear wave elastography measurements, thereby limiting the clinical validity of repeated assessments. Nevertheless, all examinations were performed by experienced radiologists using a strictly standardized acquisition protocol, and prior systematic reviews and meta-analyses have demonstrated generally acceptable reproducibility of shear wave-based elastography for renal stiffness assessment [28,29]. Finally, as a single-center study, the generalizability of these findings may be limited, and validation in larger, multicenter cohorts is warranted.

From a broader perspective, renal cortical stiffness should be regarded as a dynamic parameter influenced by multiple factors, including inflammatory activity, interstitial edema, microvascular alterations, and renal perfusion, rather than as a static marker of structural damage alone [9,27]. Accordingly, shear wave elastography measurements may vary depending on hemodynamic conditions and tissue composition, as demonstrated in previous experimental and clinical studies [30]. Emerging evidence suggests that combining elastography with other ultrasound-based techniques, such as Doppler-derived perfusion indices or contrast-enhanced ultrasound, together with relevant clinical and laboratory parameters, may provide a more comprehensive assessment of renal injury. Several recent studies have shown that such multimodal approaches can improve both diagnostic accuracy and prognostic stratification in various forms of kidney disease [18,29,31]. To further enhance the diagnostic and predictive value of elastography-based assessments in vasculitic AKI, larger prospective studies incorporating longitudinal measurements and integrated imaging–clinical models may be beneficial.

## 5. Conclusions

In conclusion, this study suggests that 2D shear wave elastography may serve as a useful noninvasive adjunctive tool for differentiating vasculitic from non-vasculitic acute kidney injury at presentation. In addition, although exploratory in nature, the observed association between baseline renal cortical stiffness and unfavorable long-term renal outcomes in patients with IgAVN is noteworthy and may provide a basis for future studies. Further prospective validation studies in larger cohorts supported by histopathological correlation are required to extend these findings and clarify the clinical role of elastography in vasculitic acute kidney injury.

## Figures and Tables

**Table 1:** Etiologies of acute kidney injury across the study groups.

Vasculitic AKI	Non-Vasculitic AKI
N = 22	N = 18
Lupus vasculitis (1)	Atypical HUS (1)
Microscopic polyangiitis (1)	C3 glomerulopathy (1)
Sjögren-associated vasculitis (1)	Pyelonephritis (2)
Rheumatoid vasculitis (1)	Unknown etiology (3)
Granulomatous polyangiitis (4)	Drug-associated TIN (5)
Immunoglobulin A vasculitis (14)	Prerenal AKI (6)

AKI: Acute Kidney Injury, HUS: Hemolytic Uremic Syndrome, TIN: Tubulointerstitial Nephritis.

**Table 2:** Comparison of clinical, ultrasonographic and elastographic parameters among the groups.

	Overall N = 60 ^1^	Vasculitic AKI N = 22 ^1^	Non-Vasculitic AKI N = 18 ^1^	Healthy Group N = 20 ^1^	*p*-Value ^2^
Age (years)	38 ± 12	39 ± 12	41 ± 14	36 ± 11	0.41
Female	32 (53)	11 (50)	10 (56)	11 (55)	0.92
Body mass index (kg/m^2^)	25 ± 2.9	24.6 ± 3.4	26.1 ± 2.4	24.6 ± 2.6	0.08
Serum creatinine (mg/dL)	1.53 ± 1.54	1.43 ± 1.69	2.66 ± 1.53	0.63 ± 0.15	<0.01
Glomerular filtration rate (mL/min/1.73 m^2^)	85 ± 47	85 ± 41	35 ± 25	129 ± 5	<0.01
Left kidney craniocaudal length (mm)	110 ± 12	111 ± 10	117 ± 10	104 ± 11	0.01
Left kidney parenchymal thickness (mm)	16 ± 3.5	15.5 ± 3.5	16.7 ± 3.3	15.8 ± 3.8	0.43
Left kidney cortical stiffness (kPa)	7.33 ± 2.45	9.37 ± 1.96	6.79 ± 2.26	5.56 ± 1.16	<0.01
Right kidney craniocaudal length (mm)	110 ± 10	112 ± 11	114 ± 11	109 ± 8	0.05
Right kidney parenchymal thickness (mm)	14.8 ± 3.8	15.3 ± 3.5	16 ± 4.5	13.3 ± 3.1	0.05
Right kidney cortical stiffness (kPa)	7.3 ± 2.55	9.63 ± 2.31	6.43 ± 1.71	5.51 ± 1.15	<0.01
Mean kidney cortical stiffness (kPa)	7.31 ± 2.37	9.5 ± 1.9	6.61 ± 1.89	5.53 ± 0.92	<0.01

^1^ Mean ± SD; n (%), ^2^ Welch’s ANOVA; Pearson’s Chi-squared test. AKI: Acute Kidney Injury, kPa: Kilopascal.

**Table 3:** Baseline clinical and elastographic findings in IgA vasculitis patients stratified by one-year renal outcomes.

	Patients with Unfavorable Renal Outcomes N = 3	Patients with Favorable Renal Outcomes N = 9	*p*-Value
Left kidney cortical stiffness kPa, median (min–max)	11.2 (10–12.6)	9.6 (5.6–11.8)	0.06
Right kidney cortical stiffness kPa, median (min–max)	12.3 (10.5–14)	8.5 (5.6–13)	0.049
Mean cortical stiffness of both kidneys, median (min–max)	11.2 (10.8–13.3)	9.1 (5.6–11.2)	0.046
Age, years	47 (37–57)	32 (21–47)	0.06
Baseline serum creatinine, mg/dL median (min–max)	0.5 (0.4–0.7)	0.8 (0.5–1.5)	0.06
Baseline proteinuria level, mg/day median (min–max)	730 (420–2200)	336 (280–1080)	0.22
Baseline BMI, kg/m^2^, median (min–max)	25.7 (23.5–26.9)	24.2 (22.5–25.7)	0.40

kPa: Kilopascal, BMI: Body Mass Index. Due to the small number of patients in the unfavorable outcome group, continuous variables are presented as median (min–max).

## Data Availability

The data can be provided by the authors upon request, with the removal of identifiable information and the anonymization of dates.

## Supplementary Materials

The following supporting information can be downloaded at https://www.mdpi.com/article/10.3390/jcm15031122/s1, Figure S1. Flow diagram of patient inclusion, exclusion, and follow-up across the two phases of the study; Table S1. Immunosuppressive treatment characteristics during 12-month follow-up in patients with IgA vasculitis nephritis; Table S2. Renal histopathological findings and cortical stiffness measurements in patients who underwent kidney biopsy; Table S3. Multivariable linear regression analysis of factors associated with mean renal cortical stiffness.

## Abbreviations

The following abbreviations are used in this manuscript:

AKI	Acute Kidney Injury
2D-SWE	Two-Dimensional Shear-Wave Elastography
IgAVN	Immunoglobulin A Vasculitis Nephritis
kPa	Kilopascal
ANA	Antinuclear Antibody
ENA	Extractable Nuclear Antigen
PR-3 ANCA	Anti-proteinase 3 Antibody
MPO-ANCA	Anti-myeloperoxidase Antibody
V-AKI	Vasculitic Acute Kidney Injury
NV-AKI	Non-vasculitic Acute Kidney Injury
KDIGO	Kidney Disease: Improving Global Outcomes
eGFR	Estimated Glomerular Filtration Rate
ROI	Region of Interest
ROC	Receiver Operating Characteristic
HUS	Hemolytic Uremic Syndrome
TIN	Tubulointerstitial Nephritis
SD	Standard Deviation
BMI	Body Mass Index
IgA	Immunoglobulin A
