# Supplementary material for: Renal Shear Wave Elastography for Differentiating Vasculitic and Non-Vasculitic Acute Kidney Injury
Source: J Clin Med. 2026 Jan 31;15(3):1122. doi: 10.3390/jcm15031122 (PMC12898426; doi:10.3390/jcm15031122)
Supplement: Supplementary file 1 [file jcm-15-01122-s001.zip › jcm-4086017-supplementary.pdf]

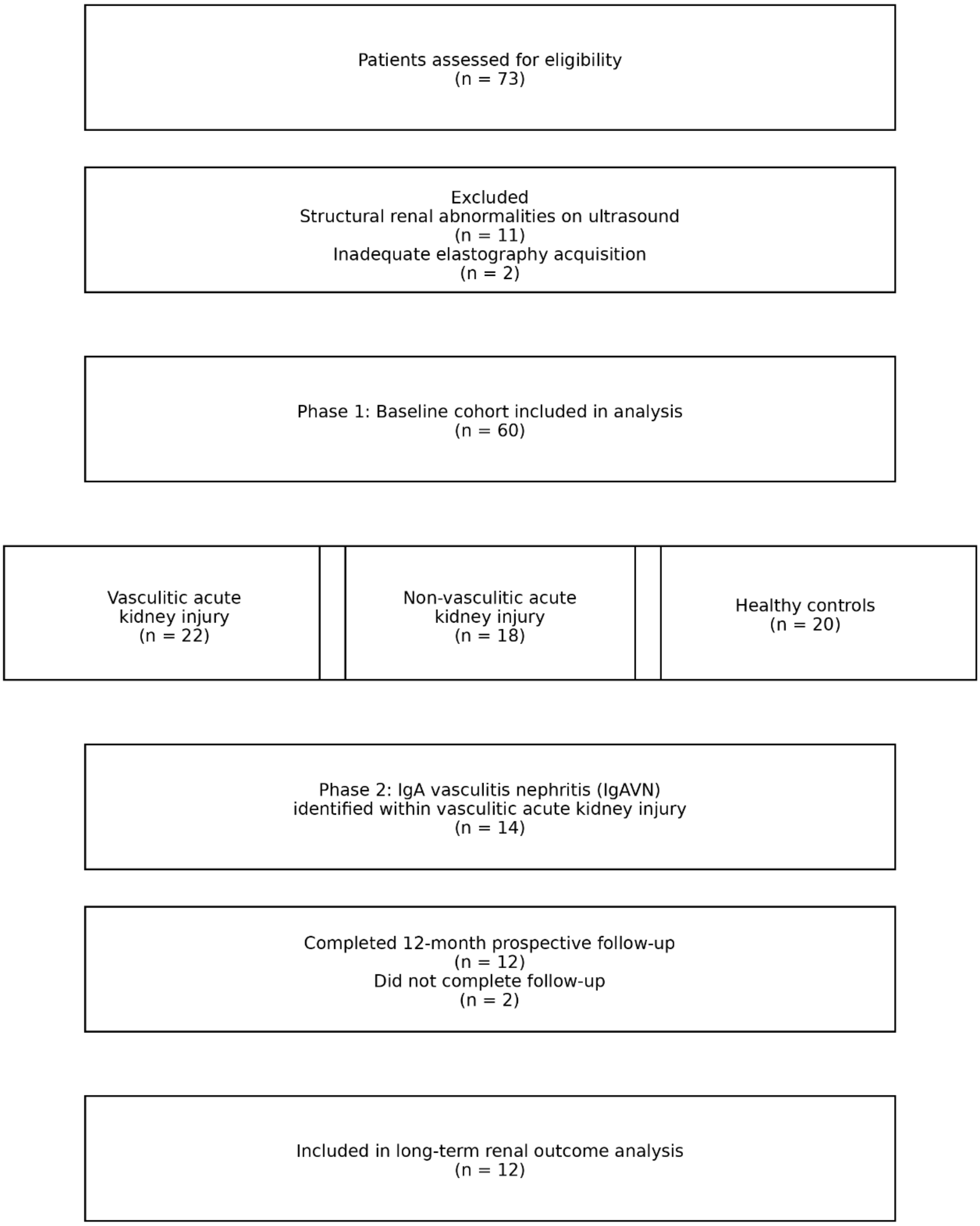

Supplementary Figure S1. Flow diagram of patient inclusion, exclusion, and follow-up across the two phases of the study.

Supplementary Table S1. Immunosuppressive treatment characteristics during 12-month follow-up in patients with IgA vasculitis nephritis

	**Patients with unfavorable renal outcomes N=3**	**Patients with favorable renal outcomes N=9**
Initial pulse steroid (≥ 250 mg/day methylprednisolone), mg, median (min-max)	2 (66.7)	3 (33.3)
Steroid sparing agent, n/N (%)		
None	0 (0)	2 (22.2)
Azatioprine	2 (66.7)	4 (44.4)
Mycophenolate mofetil	0 (0)	2 (22.2)
Cyclophosphamide	1 (33.3)	1 (11.1)
Totally steroid exposure (prednisolone equivalent), gr, median (min-max)	3.2 (1.5-4.7)	3.4 (2.9-4.7)

Mg: miligram, gr: gram, * Due to the small number of patients with unfavorable renal outcomes, treatment data are presented descriptively without formal statistical comparison.

Supplementary Table S2. Renal histopathological findings and cortical stiffness measurements in patients who underwent kidney biopsy

	Diagnosis	Histopathological Findings	Mean cortical stiffness of both kidneys (kPa)
Case 1	IgAVN	A total of 31 glomeruli; cellular crescents in 1 glomeruli; no fibrinoid necrosis; no sclerotic glomeruli; interstitial fibrosis/tubular atrophy <10 %; glomerular and mesangial IgA and C3 deposition in IF; Oxford classification M0E10S0T0C1	10.65
Case 2	RV	A total of 26 glomeruli; cellular crescents in 6 glomeruli; fibrocellular crescents in 3 glomeruli; fibrinoid necrosis in 5 glomeruli; no sclerotic glomeruli; interstitial fibrosis and tubular atrophy <10%; pauci-immune IF pattern	8.56
Case 3	SLE Vasculitis	A total of 16 glomeruli; cellular crescents in 4 glomeruli; fibrinoid necrosis in 2 glomeruli; no interstitial fibrosis and tubular atrophy; no full-house pattern in IF with rare vessel wall IgG/C3 deposition	11.91
Case 4	IgAVN	A total of 48 glomeruli; no cellular or fibrocellular crescents; no fibrinoid necrosis; no sclerotic glomeruli; interstitial fibrosis/tubular atrophy <10 %; glomerular and mesangial IgA and C3 deposition in IF; Oxford classification M0E0S0T0C0	9.22
Case 5	IgAVN	A total of 31 glomeruli; cellular crescents in 1 glomerulus; no fibrinoid necrosis; no sclerotic glomeruli; no interstitial fibrosis/tubular atrophy; glomerular and mesangial IgA, fibrinogen and C3 deposition in IF; Oxford classification M1E1S0T0C1	8.25
Case 6	IgAVN	A total of 10 glomeruli; cellular crescent in 2 glomeruli; no fibrinoid necrosis, no sclerotic glomeruli, interstitial fibrosis/tubular atrophy <10%; glomerular and mesangial IgA and C3 deposition in IF; Oxford classification M1E0S0T0C1	10.61
Case 7	IgAVN	A total of 27 glomeruli; cellular crescents in 4 glomeruli; fibrocellular crescents in 1 glomerulus; fibrinoid necrosis in 4 glomeruli; no sclerotic glomeruli; interstitial fibrosis/tubular atrophy <10%; glomerular and mesangial IgA and IgG deposition in IF; Oxford classification M0E0S0T0C1	6.54
Case 8	IgAVN	A total of 27 glomeruli; cellular crescents in 3 glomeruli; no fibrinoid necrosis; no sclerotic glomeruli; no interstitial fibrosis/tubular atrophy; glomerular and mesangial IgA and C3 deposition in IF; Oxford classification M1E1S0T0C1	13.3
Case 9	IgAVN	A total of 47 glomeruli; fibrocellular crescent in 2 glomeruli; no fibrinoid necrosis, 7 sclerotic glomeruli, interstitial fibrosis/tubular atrophy <10%; glomerular and mesangial IgA and IgG deposition in IF; Oxford classification M1E1S1T0C1	8.88
Case 10	IgAVN	A total of 26 glomeruli; fibrocellular crescent in 2 glomeruli, no fibrinoid necrosis, no sclerotic glomeruli, no interstitial fibrosis/tubular atrophy; glomerular and mesangial IgA, C3 and fibrinogen deposition in IF; Oxford classification M0E0S0T0C1	5.64
Case 11	IgAVN	A total of 15 glomeruli; no cellular or fibrocellular crescents; no fibrinoid necrosis, no sclerotic glomeruli, interstitial fibrosis/tubular atrophy <10%; glomerular and mesangial IgA and C3 deposition in IF; Oxford classification M1E0S0T1C0	11.22
Case 12	IgAVN	A total of 13 glomeruli; no cellular or fibrocellular crescents; no fibrinoid necrosis, no sclerotic glomeruli, no interstitial fibrosis/tubular atrophy; glomerular and mesangial IgA, IgM and C3 deposition in IF; Oxford classification M0E0S0T0C0	9.19
Case 13	IgAVN	A total of 18 glomeruli; cellular crescents in 2 glomeruli; fibrinoid necrosis in 1 glomerulus; 2 sclerotic glomeruli, no interstitial fibrosis/tubular atrophy; glomerular and mesangial IgA and C3 deposition in IF; Oxford classification M1E10S1T0C1	9.82
Case 14	IgAVN	A total of 13 glomeruli; no cellular or fibrocellular crescents; no fibrinoid necrosis; no sclerotic glomeruli, no interstitial fibrosis/tubular atrophy; glomerular and mesangial IgA and C3 deposition in IF; Oxford classification M1E0S0T0C0	6.95

IF: Immunofluorescence, kPa: Kilopascal, MPA: Microscopic polyangiitis, RV: Rheumatoid vasculitis, GPA: Granulomatosis with polyangiitis, SLE: Systemic lupus erythematosus, IgAVN: Immunoglobulin A vasculitis nephritis, Oxford Classification M: Mesangial hypercellularity; E: Endocapillary hypercellularity; S: Segmental glomerulosclerosis; T: Tubular atrophy / interstitial fibrosis; C: Crescents

Supplementary Table S3. Multivariable linear regression analysis of factors associated with mean renal cortical stiffness.

Variable	β (kPa)	95% CI	P -value
Vasculitic acute kidney injury	3.13	1.68 to 4.58	***<0.01***
Left kidney craniocaudal length (mm)	0.013	-0.051 to 0.077	0.68
Body mass index (kg/m^2)	0.024	-0.191 to 0.238	0.82
Baseline serum creatinine (mg/dL)	0.105	-0.300 to 0.510	0.60

Model adjusted for body mass index (BMI), baseline serum creatinine, and left kidney length. The dependent variable was mean renal cortical stiffness, expressed in kilopascals (kPa)
